# Biological therapies for premature ovarian insufficiency: what is the evidence?

**DOI:** 10.3389/frph.2023.1194575

**Published:** 2023-09-07

**Authors:** Melpomeni Moustaki, Adamantia Kontogeorgi, Gkalia Tsangkalova, Haralampos Tzoupis, Antonis Makrigiannakis, Andromachi Vryonidou, Sophia N. Kalantaridou

**Affiliations:** ^1^ Department of Endocrinology and Diabetes Center, Hellenic Red Cross Hospital, Athens, Greece; ^2^ Serum IVF Fertility Center, Athens, Greece; ^3^ Department of Obstetrics and Gynecology, University of Crete Medical School, Heraklion, Greece; ^4^ 3rd Department of Obstetrics and Gynecology, National and Kapodistrian University of Athens, Medical School, Athens, Greece

**Keywords:** Premature Ovarian Insufficiency, stem cell therapy, exosomes, biological therapies, HRT, PRP, in vitro activation, microRNAs

## Abstract

Premature Ovarian Insufficiency (POI) is a multi-factorial disorder that affects women of reproductive age. The condition is characterized by the loss of ovarian function before the age of 40 years and several factors have been identified to be implicated in its pathogenesis. Remarkably though, at least 50% of women have remaining follicles in their ovaries after the development of ovarian insufficiency. Population data show that approximately up to 3.7% of women worldwide suffer from POI and subsequent infertility. Currently, the treatment of POI-related infertility involves oocyte donation. However, many women with POI desire to conceive with their own ova. Therefore, experimental biological therapies, such as Platelet-Rich Plasma (PRP), Exosomes (exos) therapy, In vitro Activation (IVA), Stem Cell therapy, MicroRNAs and Mitochondrial Targeting Therapies are experimental treatment strategies that focus on activating oogenesis and folliculogenesis, by upregulating natural biochemical pathways (neo-folliculogenesis) and improving ovarian microenvironment. This mini-review aims at identifying the main advantages of these approaches and exploring whether they can underpin existing assisted reproductive technologies.

## 1. Introduction

Premature Ovarian Insufficiency (POI) is a condition characterized by the loss of ovarian function in women before 40 years of age (1). POI occurs in the setting of ovarian follicles dysfunction or depletion (2), leading to oligo/amenorrhea, hypergonadotropic hypogonadism and infertility (3, 4). According to the European Society for Reproduction and Embryology (ESHRE), the diagnostic criteria for POI include oligo/amenorrhea for at least 4 months and follicle stimulating hormone (FSH) level >25 iu/L, in 2 occasions, at least 4 weeks apart before the age of 40 years (4). The assessment of ovarian reserve by biochemical indicators like antral follicle count (AFC) and anti-mullerian hormone (AMH) serum levels (5) is not necessary in order to establish diagnosis, but might be informative when fertility is to be sought (1). The relevant symptomatology involves vasomotor symptoms (night sweats and hot flushes), vulvovaginal atrophy, vaginal dryness and dyspareunia, insomnia, mood disturbances, cognitive problems such as memory issues, fatigue, loss of libido and weight gain (1), which, undoubtedly compromise the quality of life and sexual function of affected women (4). Furthermore, the condition bears long-term sequalae of decreased bone mass, increased cardiovascular risk, and decreased life expectancy (4).

Over the last decade, various studies report that 1%–3.7% of the female population worldwide suffers from POI (6–8). The aetiology of the disease is multifactorial, encompassing genetic defects (Turner's syndrome, Fragile X messenger ribonucleoprotein 1, premutation, galactosemia), autoimmunity, infectious diseases (tuberculosis, mumps, human immunodeficiency virus, infection), exposure to smoking or environmental endocrine disruptors, and iatrogenic causes (chemotherapy, radiotherapy, pelvic surgeries, embolism of uterine arteries); nevertheless, the aetiology remains unknown in the vast majority (∼75%) of cases (idiopathic POI) (1, 9).

The mainstay of POI treatment is hormone replacement therapy (HRT) (1, 4, 10) with oestrogen and progestin in continuous or cyclic regimens. The treatment is usually continued until the age of normal natural menopause (1). While HRT alleviates POI symptomatology and protects women from long-term effects of hypoestrogenism (11, 12), it has shown little to no effect in addressing infertility, which has been described as the most devastating aspect of POI by patients (11). Moreover, conventional ovarian stimulation protocols have been used for ovulation induction in women with POI with poor outcomes (1). Up to now, the only validated assisted reproduction technique for these women is oocyte donation (4).

Nevertheless, women with POI have 5%−10% chance of spontaneous pregnancy (4). This is ascribed to the maintenance of intermittent ovarian function (2). Actually, histological data demonstrate the presence of primordial follicles (Pfs) in 50% of the ovaries of women with POI (13). This notion has stimulated various attempts of ovarian rejuvenation, aiming at inducing the differentiation of ovarian stem cells (OSCs) to oocytes, transitioning Pfs to primary follicles, and delaying the apoptosis of existing follicles. The current review focuses on novel biological strategies that can be employed for the treatment of infertility in women with POI, aiming to explore if their efficacy is superior compared to traditional treatment, based on evidence originating from clinical and preclinical studies. These experimental protocols involve: (i) platelet-rich plasma (PRP), (ii) exosome therapy, (iii) *in vitro* activation (IVA), (iv) stem cells therapy, (iv) microRNAs and (v) mitochondrial targeting therapies (14).

## 2. Methodology

**Figure 1:**
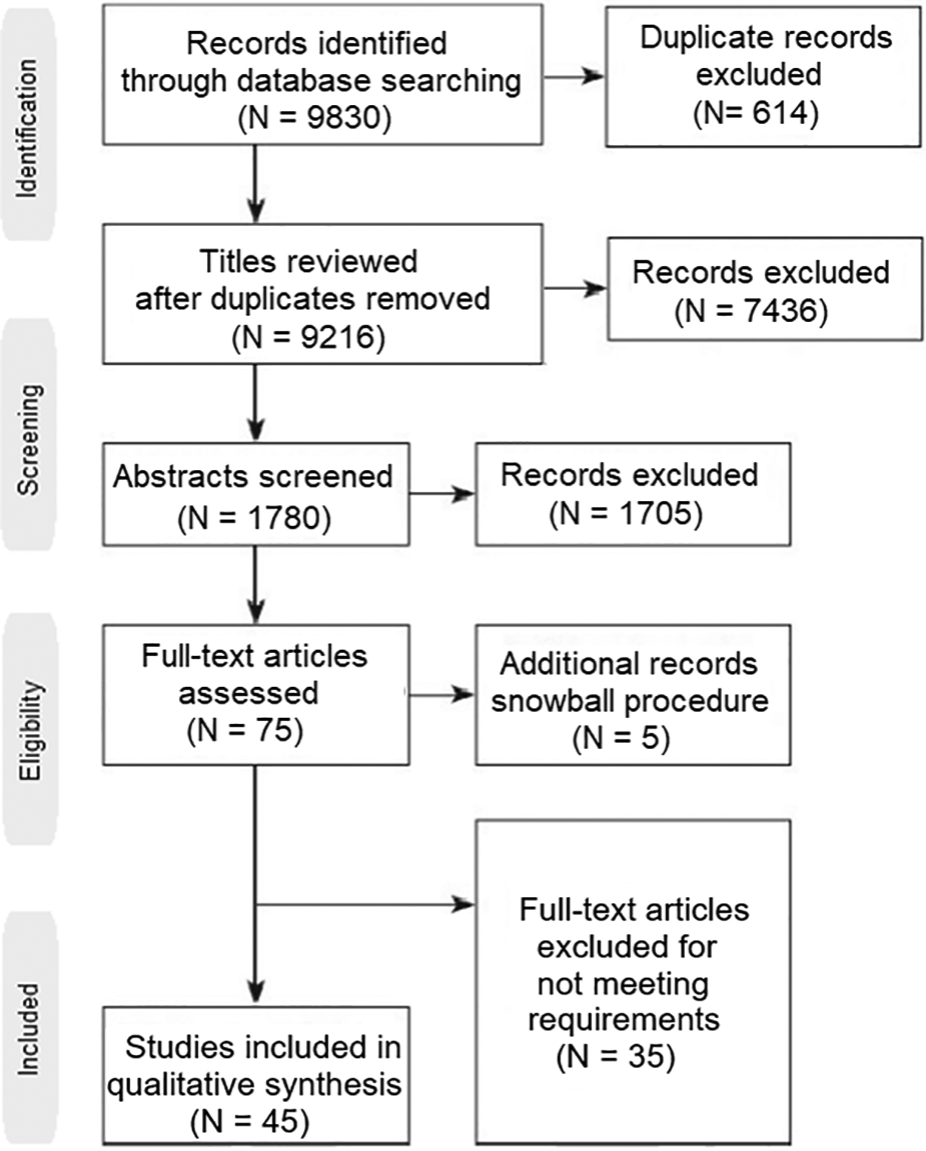
PRISMA diagram describing the selection process for studies included.

The current review has been conducted by following all PRISMA (Preferred Reporting Items for Systematic Reviews and Meta-Analyses) guidelines. The authors performed a literature search in three medical databases, Pubmed, Embase and Cochrane Library for the last 10 years. The key-words used for the search were: “premature ovarian insufficiency”, “POI”, “premature ovarian failure”, “POF”, “platelet-rich plasma”, “PRP”, “in vitro activation”, “IVA”, “stem cells”, “exosomes”, “mitochondrial replacement therapy” and “microRNAs”. The query used in all three databases was: (“premature ovarian insufficiency” OR “POI” OR “premature ovarian failure” OR “PRP”) AND (“platelet-rich plasma” OR “PRP” OR “in vitro activation” OR “IVA” OR “stem cells” OR “exosomes” OR “mitochondrial replacement therapy” OR “microRNAs”). Following the snowball procedure, only papers referring to applications in POI were selected, both original and review papers. Based on the references included in the review papers, more original papers were retrieved, leading to a total of forty five original papers and twenty review papers on the topic (Figure 1).

## 3. Cell-Free therapies

### 3.1. Platelet-rich plasma (PRP)

Platelet-rich Plasma (PRP) is an autologous blood derivative, containing greater concentration of platelets (PLTs) (3- to 5-fold) when compared to peripheral blood (15). PRP has a 5- to 10-fold higher concentration of growth factors (GFs) (>800 types) than peripheral blood (16, 17). These factors, principally subclassified as chemokines, mitogens and antigens, are released following PRP activation by various agonists and act in a paracrine manner, mediating tissue regeneration and homeostasis (18, 19). The above effects are supported by induction of cell migration, chemotaxis, angiogenesis, keeping balance between proliferation and apoptosis, and control of inflammation and oxidative stress (15, 19). As a matter of fact, PRP's effects are not specific but support the needs of each tissue in which is applied. Owing to this adaptive capacity, PRP has been increasingly used in a variety of clinical settings, including dentistry, sports medicine, dermatology/cosmetology, ophthalmology, neurology as well as in reproductive medicine (20).

According to preliminary data in rat models of POI, intraperitoneal or intraovarian administration of PRP is demonstrated to increase ovarian cortex volume, pre-antral follicle count, and antral follicle diameter (21), while decreasing the atretic follicle count (22). Furthermore, their litter count is significantly increased post PRP (22). The above, favourable results of PRP are replicated in mammals with ovarian hypofunction; in one study, 80% of PRP-treated cows show increased progesterone level at 4 weeks post-PRP and clinical pregnancy after artificial insemination (AI) is achieved in all of them (23).

Over the last four years, emerging data from few clinical studies in women with POI support the efficacy of PRP in ovarian rejuvenation. In particular, intra-ovarian PRP administration is shown to restore the menstrual cycle in 22%–60% of women in cohort studies (24, 25) and up to 100% in case reports or case series (26–28), as well as to induce spontaneous ovulation (28). These effects are accompanied by increase in oestradiol (E2) (24) and decrease in luteinizing hormone (LH) levels (24, 26). Apart from hormonal recovery, PRP appears to improve ovarian reserve parameters, such as AMH and AFC (24, 26, 27, 29); this is also reflected at the decreased level of follicle stimulating hormone levels (FSH) (24, 26–28). Regarding pregnancy outcomes, PRP is shown to increase the rates of spontaneous conception to 7.4%–10% (24, 29) in two cohort studies; nevertheless, 0% rate of spontaneous conception is reported in another study (25). Importantly, PRP-induced increase in AFC leads to successful oocyte retrieval post-ovarian stimulation and allows *in vitro* fertilization (IVF) procedures to take place (27–29). According to the only relevant, prospective cohort study in 311 women, there is a 26.4% possibility of embryo formation and a further 22.8% possibility of pregnancy following embryo transfer (ET) (29). Last but not least, live birth rate (LBR) ranges between 69%–100% (24, 29) in two cohort studies, with no difference between natural and IVF conception in one of them (24); however, it should be highlighted that the real numbers of pregnancies achieved in both studies are rather small (36 and 3 respectively), especially in the one showing LBR of 100% (Table 1). Looking for prognostic factors, residual baseline ovarian activity assessed by AMH, AFC and FSH (24, 29), as well as short duration of amenorrhea seem to predict a positive response. Concerning the latter, it is worth mentioning that in the study showing 0% spontaneous pregnancy, the mean duration of amenorrhea is 8 years (25), while in the study in which the respective rate is 10%, the mean duration of amenorrhea is 10 months (24). Interestingly, age does not seem to affect PRP outcomes in POI patients (24) and this is consistent with corresponding results in patients with poor ovarian response (POR) (39).

**Table 1:** Clinical studies examining the effect of biological therapies in women with POI.

Platelet-Rich-Plasma (PRP)						
Study	Study design	*N*	Subjects	Intervention	Follow-up	Outcomes
Sfakianoudis et al. (2018) (27)	Case report	1	40 y, age at POI diagnosis = 35 y, abandoned HRT for 4 m, amenorrhea	PRP (RegenACR-C kit, concentration 9 × 10^5^/μl, 4 ml bilaterally, TV, US guided-intramedullary, multifocally). Followed by natural cycle IVF-ICSI	N/A	Resumption of menses at 6w, ↓ FSH, ↑ AMH, Biochemical pregnancy resulting into spontaneous abortion at 5w
Pantos et al. (2019) (26)	Case series	2	27 & 40 y, amenorrhea > 1y, previous failed IVF, not receiving HRT	PRP (4 ml bilaterally, TV, US-guided, intramedullary, multifocally) Invited to conceive naturally	N/A	Resumption of menses, ↓ FSH, LH ↑ AMH, AFC, clinical pregnancy after 2 & 4 m.
Cakiroglu et al. (2020) (29)	Prospective, cohort study	311	POI (ESHRE 2016)^a^, 24–40 y, infertility history of >1 y	PRP (T-Biotechnology Laboratory kit, Bursa, Turkey, 2–4 ml, TV, US-guided, at subcortical and stromal areas, at least in one ovary), within 10 days after completion of menstruation or randomly. 2 m allowed for spontaneous conception. Then, if not pregnant but had AFC ≥1 developed: COS + IVF ± ET	1 y	↑ AFC & AMH (d2–4)^b^, ↔ FSH, 7.4%^c^ spontaneous pregnancy, 64.8%^c^ had IVF, 26.4%^c^ developed embryos, 4%^c^ achieved pregnancy post ET (22.8% per ET), 8%^c^ cryopreserved embryos, 8%^c^ livebirth/sustained implantation (69% of both spontaneous & IVF pregnancies)
Hsu et al. (2020) (28)	Case report	1	37-y, 6m-amenorrhea, previous POR to Gn, asked to discontinue HRT for 2m	GC-activated PRP (5 ml) +Gn (1 ml consisting of 150iu FSH +75iu LH), 3 ml at each ovary, TVUS-guided, into ovarian stroma + COS (intermittent vaginal administration) + ICSI	N/A	↓FSH, spontaneous ovulation, menses resumption, oocyte yield = 6, embryos =3, twin pregnancy, preterm delivery (30w) with no documented abnormalities
Sfakianoudis et al. (2020) (24)	Prospective, cohort, pilot study	30	POI (ESHRE 2016)^a^ presenting with amenorrhea, <40 y, HRT discontinued for 6m	CG-activated PRP (RegenACR-C kit, concentration 1 × 10^6^/μl, 4 ml, bilaterally, multifocally), on random time. Invited to conceive naturally	1 y	At 3 m follow-up: 60% resumption of menses, ↓ FSH, LH & ↑ AMH, E2, AFC (d3 or monthly post PRP) 10% spontaneous pregnancies and live births
Aflatoonian et al. (2021) (25)	Prospective, cohort study	9	POI (ESHRE 2016)^a^ HRT discontinued 1 month before and after PRP	GC-activated PRP (Rooyagen, Tehran, Iran, concentration 3-5-fold higher than basal blood samples,1.5 ml, multifocal, intramedullary in each ovary) + 2nd injection (3 ml) after 3 m in case of no pregnancy, on d10 or randomly. Invited to conceive naturally	1 y	22% menstruation recovery (after 2nd PRP injection), no hormonal recovery (in 2 m), 0% spontaneous pregnancy
In vitro activation (IVA)						
Kawamura et al. (2013) (30) & Suzuki et al. (2015) (13)	Prospective, cohort study	37	POI: < 40y, with amenorrhea >4 m, FSH >35 iu/L (initial criteria: amenorrhea >1y, FSH > 40 iu/L) Mean age = 37 years	Laparoscopic ovariectomy. Ovarian cortices were dissected into strips for vitrification. Some pieces were examined histologically. After warming, strips were fragmented into smaller cubes and cultured with Akt stimulators for 2 days. Then, ovarian cubes were transplanted beneath the serosa of fallopian tubes. Then ovarian stimulation was performed (estrogen pre-treatment, ± controlled). After oocyte retrieval, ICSI was performed (Kawamura protocol)	1 y	54% had residual follicles based on histology, 24 oocytes retrieved from 6 patients, 3 pregnancies, 2 live births
Zhai et al. (2016) (31)	Prospective, cohort study	14	POI: < 40y, amenorrhea ≥ 1y, FSH > 35 iu/L in at least 2 occasions, 4w apart, E2 < 20pg/ml With previous proof of follicle development post Gn stimulation	Kawamura protocol with fresh ovarian tissue instead of frozen-thawed ones. When follicles reached the preovulatory stage, hCG treatment was initiated.	1 y	50% had residual follicles based on histology, 6 patients showed 15 follicular development waves reaching pre-ovulatory stage, 4 patients had successful oocyte retrieval and developed embryos, 1 pregnancy & live birth, 3 cryopreserved embryos
Fabregues et al. (2018) (32)	Case report	1	POI, 32y, FSH = 89.9 iu/L, AMH = 0.02 ng/ml, HRT discontinued 1 day before intervention	The removal of the ovarian cortex and autotransplantation were performed by laparoscopy in the same surgical act. Ovarian fragments were transplanted in contralateral ovary and peritoneal pocket near to the ovary. Immediately after surgery GnRH agonist together HMG injections were initiated.	N/A	Retrieval of 2 oocytes, formation of 2 embryos, pregnancy (singleton)
Stem cell (SC) transplantation						
Edessy et al. (2014) (33)	Prospective cohort study	10	POI: 26–33 y, FSH≥ 20 iu/L, normal karyotype	Autologous BMSCs (from iliac crest) transplantation into ovaries laparoscopically	6 m	10% resumption of menses, 20% focal secretory changes in endometrium Both results correlated with OCT4 expression and ESS ≥5
Chen et al. (2018) (34)	Case report	1	POI: 38y, amenorrhea for 2y	MSCs monthly, for 6 m (concentration 2 × 10^7^/ml at first time, 1 × 10^7^/ml at subsequent times), IV	N/A	↑ ovarian size, endometrial thickness & blood flow in endometrium (TV and transabdominal US)
Ding et al. (2018) (35)	Blind, RCT	14, divided in 2 groups UC-MSCs group: 6 collagen/ UC-MSCs group: 8	POI: 18–39 y, amenorrhea >1y, FSH ≥40 iu/L on 2 occasions 4–6w apart, normal sperm analysis of the partner	UC-MSCs group: allogeneic UC-MSCs (concentration 5 × 10^6^/400 μl) + HRT (group A) collagen/ UC-MSCs group: collagen/ UC-MSCs (allogeneic, UC-MSCs concentration 5 × 10^6^/400 μl, collagen concentration 5 mg/ml) + HRT (group B) TVUS-guided, unilateral intraovarian injection, up to 4 transplantations	1 y	↑ E2^d^, ↓ FSH^d^ (collagen/ UC-MSCs group), ↑ ovarian volume^d^ (UC-MSCs group), presence of blood flow in some patients of both groups at 3 m, active signs of follicle-like structures (16.7% in UC-MSCs group, 62.5% in collagen/ UC-MSCs group) 14.3% natural conception (equally among groups)
Igboeli et al. (2020) (36)	Case report (2 first cases of ongoing clinical trial)	2	POI: case 1: 36y, amenorrhea for 4y and FSH = 110 iu/L, case 2: 42y, amenorrhea, left ovariectomy, FSH = 155 iu/L	Autologous BMSCs (from iliac crest) transplantation into right ovary (4 ml) laparoscopically + 4 ml normal saline in the left ovary (control, only in case 1)	1 y	Resumption of menses (1 menstruation in 1y), ↑ E2 by 150%, improvement in menopausal symptoms ↑ovarian volume by 50% (vs. control ovary)
Mashayekhi et al. (2021) (37)	Non-randomized clinical trial, phase 1	9, divided in 3 groups (according to ADSCs amount)	POI: 20–39y, with FSH ≥25 iu/L in 2 occasions, 4w apart, amenorrhea >1y	Autologous ADSCs (subabdominal fat pads) transplantation of 5 × 10^6^, 10 × 10^6^, or 15 × 10^6^ in unilateral ovary TV in 7 cases & laparoscopically in 2 cases.	1 y	Primary outcome: no SEs/complications Secondary outcomes: 44.4% resumption of menses, ↓ FSH <25 iu/L (44.4%) without differences among groups, ↔ AFC, AMH
Zafardoust et al. (2023) (38)	Prospective cohort study	15	POI	Autologous Men-MSCs	1 y	100% improvement of menopausal symptoms, 2.6% resumption of menses, ↑AFC, E2, ↓FSH, LH, ↔ AMH

AFC, antral follicle count; ADSCs: adipose-derived stromal cells; AMH, anti-mullerian hormone; BMSCs, bone marrow-derived mesenchymal stem cells, collagen/US-MSCs, umbilical cord mesenchymal stem cells on a collagen scaffold; COS: controlled ovarian stimulation; d, day of menstrual cycle, ESS: Edessy stem cell score, ESHRE, European Society of Human Reproduction and Embryology; E2, oestradiol, FSH, follicle-stimulating hormone; Gn, gonadotropin; GnRH, gonadotropin releasing hormone, HMG, human menopausal gonadotropin; HRT, hormone replacement therapy; ICSI, intracytoplasmic sperm injection; IV, intravenously, IVF, *in vitro* fertilization; LH, luteinizing hormone; m, months; Men-MSCs: menstrual blood-derived mesenchymal stem cells; MSCs, mesenchymal stem cells; N/A, not applicable; OCT4, octamer-binding transcription factor 4;POI, primary ovarian insufficiency; POR, poor ovarian response; PRP, platelet-rich plasma; RCT, randomized controlled trial; TV, transvaginal; TVUS: transvaginal ultrasound, US, ultrasound; US-MSCs, umbilical cord mesenchymal stem cells; y, years, w, weeks.

^a^ ESHRE criteria for POI diagnosis: oligo/amenorrhea for at least 4 m and FSH > 25 iu/L in 2 occasions, 4 weeks apart, age <40 years.

^b^ in case of absence of menstruation, menses was hormonally induced.

^c^ percentage over the total no. of participants.

^d^ compared to baseline.

In all the above clinical studies, the route of intraovarian PRP administration is transvaginal, via 17G−18G lumen needle (24–29). The procedure is ultrasound-guided and resembles the transvaginal paracentesis during oocyte pick up (24), performed under minimal sedation (24–27, 29). In most studies, PRP is delivered intramedullary, at multiple sites, apart from two studies, in which it is also diffused in the subcortical layers (27, 29).

The above observed improvement of ovarian function in POI in clinical and preclinical studies by GFs contained in PRP involves three main events. Firstly, the recruitment of uncommitted OSCs to differentiate into *de novo* oocytes (*de novo* oogenesis) (15, 19), secondly, the activation of dormant Pfs and support of each step of folliculogenesis from Pf to pre-ovulatory follicle, and, thirdly, the decrease in apoptosis (atresia) of existing follicles (18). The Pf to primary follicle transition is mediated by platelet-derived growth factor (PDGF), transforming growth factor beta (TGF-β), hepatocyte growth factor (HGF), connective tissue growth factor (CTGF) and shingosine-I-phosphate (SIP) (15, 19, 40). Further follicular proliferation and maturation is facilitated by endothelial growth factor (EGF), fibroblast growth factors (FGFs), growth differentiation factor 9 (GDF-9) and bone morphogenic proteins (BMPs) (15, 18, 19). TGF-β also participates in the crosstalk amongst thecal, granulosa and germ cells (19), which has been indicative of playing a crucial role in the development of the primary oocytes and their fertilization capacity (41). Moreover, insulin-like growth factors (IGFs) and serotonin stimulate steroidogenesis (15, 19). Finally, vascular endothelial growth factor (VEGF) exerts protection from apoptosis and oxidative stress and has a pivotal role in angiogenesis (15, 19). Indeed, enhanced vascular density, accompanied by increased expression of angiogenesis-related transcripts, namely angiopoietin 2 (ANGPT2) and kinase insert domain receptor (KDR), have been documented post PRP in rat ovarian tissue ex vivo (22).

Up to now, no adverse effects of PRP have been reported in either of the above clinical studies in POI or in similar studies in women with POR or low ovarian reserve (LOR) (42–51). However, it should be noted that the majority of these studies are not controlled and have short-term follow-up (maximum 1 year). The only controlled clinical study in women with LOR, demonstrated no difference in miscarriage and LBR between PRP and control group (51). Beyond reproductive medicine, minor side effects such as hyperpigmentation, local pain, irritation, erythema and swelling around injection sites have been reported in dermatology applications (15, 52). The most serious adverse effect, reported so far, is one case of unilateral irreversible blindness following periocular PRP administration due to ophthalmic artery occlusion with concomitant brain infarction (53). To prevent thrombotic episodes, thrombophilic disorders and/ or use of anti-coagulants, as well as malignancy are among the exclusion criteria of most of the conducted studies; however, it would be prompt to consider further parameters such as smoking, recent infection or inflammatory disease and use of combined oral contraceptives, which might also induce a hypercoagulable state, as contraindications. Regarding the risk of infection, microbial growth due to contamination of dermal microbes has been demonstrated in one study, yet without leading to infection (54). In this study, the samples where kept at 35°C for 7 days (54), therefore it is imminent that PRP should be prepared and administered under sterilized conditions, ideally immediately after its preparation. To the best of our knowledge, there is no reported case of malignancy ascribed to PRP administration. On the contrary, its use has been proven safe in oncological patients at 30–45 months follow-up (55, 56), while, surprisingly it is shown to lower the recurrence of fibrosarcoma when applied post-surgery in an animal model (57). Last but not least, PRP is autologous, eliminating the risks of immunogenicity.

### 3.2. Exosomes (exos) therapy

Exosomes (exos) are extracellular vesicles released, principally from mesenchymal stem cells (MSCs), ranging in size from 30 nm to 150 nm (58). By releasing non-coding RNA, mRNA, growth factors and proteins, exos are active in influencing cellular communication as well as the fate of recipient cells, via regulation of proliferation and apoptosis (14, 59–62). In rodent models of POI, the administration of exos from various human MSCs, such as bone marrow-derived MSCs (BMSCs) (63), umbilical cord MSCs (UC-MSCs) (64), and adipose tissue-derived MSCs (ADSCs) (65), as well as from human embryonic stem cells (ESC) (66) and human pluripotent stem cell–mesenchymal stem cells (hiMSC) (67) is demonstrated to recover the oestrous cycle (63), to increase the hormone levels, the AMH level and the number of follicles (63, 65–67), as well as to prevent follicular atresia (63, 67) and to enhance the fertility rate by reducing the time of impregnation (64).

As demonstrated *in vitro*, the main mechanism of ovarian rejuvenation induced by exos therapy is exerted at the level of granulosa cells, the quality of which is implicated in POI pathogenesis. In particular, there is increased proliferation in parallel with decreased apoptosis, being associated with upregulation of phosphoinositide 3 kinase– protein kinase B (PI3K/Akt) (66) and B-cell lymphoma 2 (Bcl2) (68), alongside with downregulation of SMAD (65) and Bcl-2 associated X protein (Bax) (68) signalling pathways. Furthermore, various microRNAs carried in human exos, such as miR-144-5p in BMSCs (63), miR-126-3p in UC-MSCs (69), and miR-369-3p in amniotic fluid MSCs (70) are shown to inhibit granulosa cell apoptosis, via various mechanisms, including the suppression of phosphatase and tensin (PTEN) (miR-144-5p) and YY1-associated factor 2 (YAF2)/programmed cell death 5 (PDCD5)/tumor protein 53 (p53) (miR-369-3p) pathways. In addition to the effect of exos therapies on granulosa cells, the attenuation of ovarian tissue fibrosis alongside with the enhanced differentiation of theca cells, both mediated via the inhibition of TGF-β1/Smad3 signalling pathway has been shown post human UC-MSCs transplantation in POI rats, contributing to the restoration of ovarian function (71).

In conclusion, the available research data is encouraging and many authors and researchers now recognize exos treatment as a potential treatment for POI. Exos are considered as potentially safer than stem cells (SCs) due to lack of tumorigenicity, low immunogenicity, and no ethical concerns (62). However, in order to be considered for POI patients, their efficacy and safety needs to be assessed in human clinical trials.

## 4. In vitro activation (IVA)

**Figure 2:**
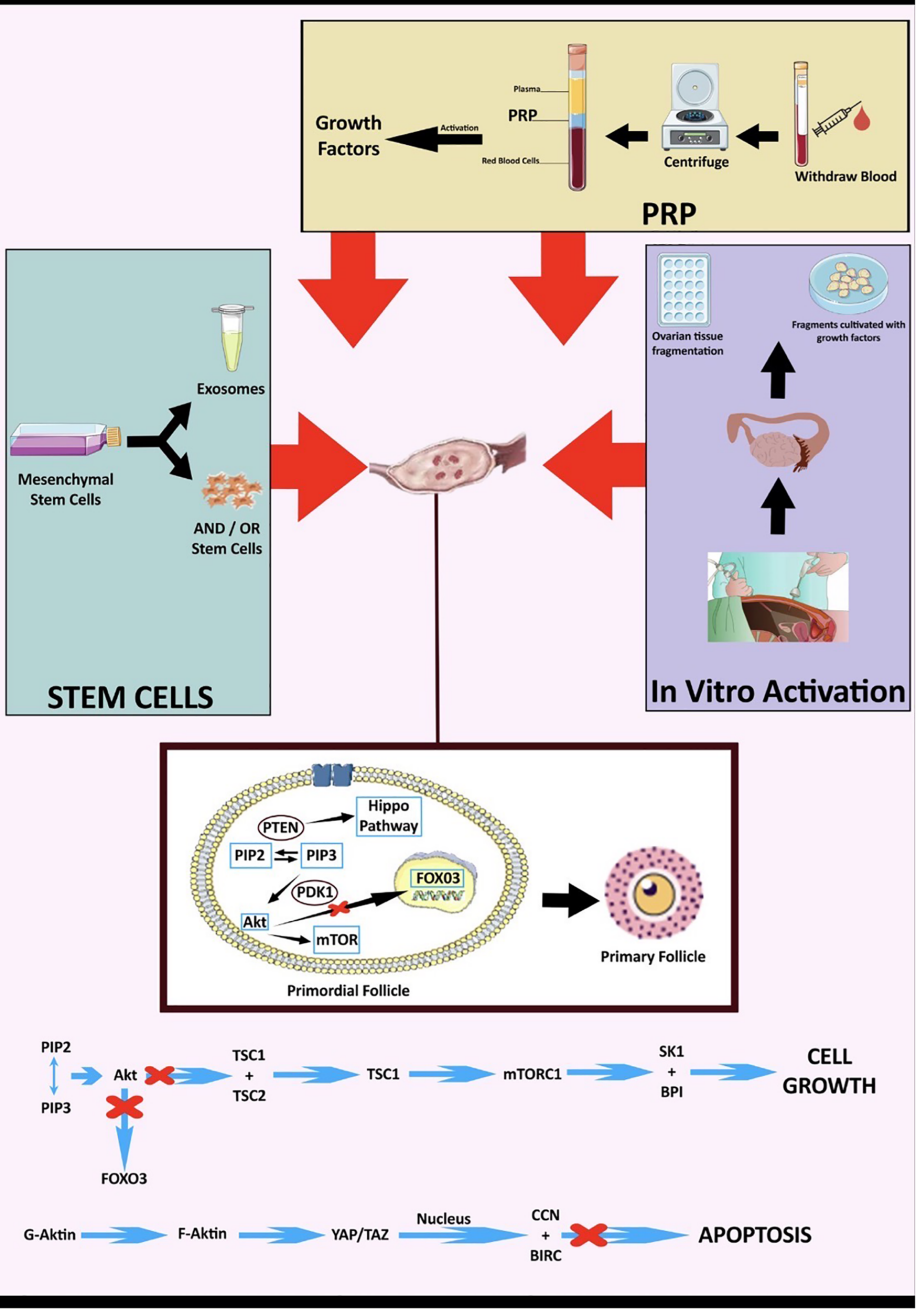
Potential mechanisms for biological therapies in POI.

Pfs activation mechanisms, in normal Pfs and dormant Pfs, have been studied mainly in animal models. Only two studies based on human cells exist. The reported studies have isolated three main signalling pathways, involved in Pfs activation, namely the PTENâ€“PI3Kâ€“Akt/transcription factor forkhead box O-3 (FOXO3), the mammalian target of rapamycin complex 1 (mTORC1), and Hippo pathways (14) (Figure 2). To simulate *in vitro* the action of the PTEN–PI3K–Akt/FOXO3 and mTORC1 pathways, the use of Akt stimulators is required, while ovarian fragmentation contributes to suppression of the Hippo pathway, and concomitant activation of Pfs (30). During Pfs activation by the PTEN–PI3K–Akt/FOXO3, the tyrosine kinase receptor boosts PI3K activity which, in turn, activates Akt signalling. PTEN–PI3K–Akt/FOXO3 activation induces mTORC1 and subsequently promotes Pfs survivability (72). On the other hand, the Hippo signalling pathway regulates the size of organs, through the inactivation of the transcriptional PDZ-binding motif (TAZ) signalling and the Yes-associated protein 1 (YEP). YEP and TAZ signalling, in turn, regulate the expression of intercellular signal proteins promoting Pfs growth (73).

In 2013, Kawamura et al. (30) combined the knowledge regarding the activation mechanisms of Pfs and applied them to the clinical practice of IVF techniques. Thus, *in vitro* activation (IVA) procedure was performed for the first time in patients with POI. In this first clinical study, ovarian tissue was obtained, histological detection of residual Pfs was performed, and, following ovarian fragmentation, tissue treatment with Akt stimulating drugs was employed. The tissue pieces, after their processing, were placed under the fallopian tube, via autologous tissue transplantation, and once they reached the antral stage, oocyte retrieval was performed. The above-described IVA technique was employed in two studies (13, 31) and two case reports (32, 74). The latter were limited to ovarian fragmentation without using Akt inducers. In total, the IVA process resulted in 6 pregnancies, of which 3 were successful live births, 2 miscarriages, and 1 ongoing pregnancy at the time of publication (Table 1). The most important observation, based on these studies, is that mature oocytes were retrieved only by patients whose histological examination of the removed ovary revealed primordial follicles.

## 5. Stem cell therapy

Stem cell therapies have been employed using, mainly, human pluripotent stem cells (hPSCs) and MSCs (75). Stem cell therapy can either be autologous, using the patient's own SCs, or allogeneic (SCs provided by a healthy donor). Like PRP, SCs have been applied in a variety of human diseases, including neurological disorders, pulmonary dysfunctions, metabolic/endocrine-related diseases, reproductive disorders, skin burns, cardiovascular conditions and liver disease (75, 76).

In animal models of POI, SC therapy, either autologous or allogeneic, is shown to restore hormonal secretion (E2, FSH) (77–82), ovarian weight/structure (77, 81, 83), follicle count (78, 79, 82–87), ovulation/oestrous cycle (78, 79, 82, 85) and pregnancy rates (78, 82). In these studies, a wide variety of SCs has been studied, including MSCs (77), ADMSCs (78, 84, 85), UC-MSCs (80, 81, 88), BMSCs (89, 90), human amniotic epithelial cells (hAECs) (83, 86, 87, 91), human amniotic mesenchymal cells (hAMCSs) (82, 91, 92) chorionic plate-derived mesenchymal stem cells (CP-MSCs) (79) and endometrial MSCs (eMSCs) (93). Interestingly, hAECs therapy seems to have better results compared to other SC therapies, because hAECs regulate oocyte telomerase activity (91) and differentiate into granulosa cells (87); nevertheless, no SC type is shown to differentiate into germ cells (85, 90, 92).

Clinical data on the application of MSCs transplantation in patients with POI are scarce, derived from case reports (34, 36) and small-numbered clinical trials, with up to 15 participants (Table 1). These clinical trials have used autologous BMSCs (33), allogeneic UC-MSCs (35), adipose tissue-derived stromal cells (ADSCs) (37) and menstrual blood-derived MSCs (Men-MSCs) (94). According to the reported results, MSCs induce menses resumption at 2.9%-44% (33, 37, 94) and up to 100% in one case report (36), and also improve menopausal symptoms (94). Further observed changes include increased level of E2 (35, 36, 94), decreased level of FSH (35, 37, 94) and LH (94), increased ovarian volume (34–36) and ovarian blood low (35), increased endometrial thickness and blood flow in endometrium (34), with documented shift from endometrial atrophy to secretory endometrium in biopsy (33). Nevertheless, SC therapy does not appear to improve AMH and AFC (37, 94), while, in total, there are only 2 reported cases of spontaneous pregnancies (35). In one of these cases employing allogeneic UC-MSC transplantation, microsatellite loci analysis showed the foetus to be genetically related to the mother and not to the donor. Finally, there are no clinical studies examining the effect of SC therapy in relation to IVF procedures in POI patients. Except for one case report in which MSCs were transplanted intravenously (34) in the rest of the aforementioned studies, SCs were transferred directly to the ovaries of the patients with a retrograde injection (35) or a simple injection into the parenchyma, either transvaginally (37) or laparoscopically (33, 36, 37).

The principal mechanism of SC-mediated ovarian rejuvenation is improvement of ovarian microenvironment, exerted by secretion of growth factors (VEGF, IGF-1, HGF) (84, 91), transcription factors (octamer-binding transcription factor 4, OCT4, homeobox protein NANOG) (91) and enzymes such as heme oxygenase 1 (88). Subsequently, there is stimulation of intracellular pathways such as Jun N-terminal kinase (JNK)/Bcl2 (88) and Janus activated kinase (JAK)/signal transducers and activators of transcription (STAT), enhanced Bcl2 (92), VEGF(92), VEGF receptor 1 (VEGFR1) (86) and VEGF receptor 2 (VEGFR2) (86) expression and inhibition of Bax expression (92). The above effects promote cell proliferation and angiogenesis, and decrease apoptosis and inflammation (83, 84, 86). In addition, many studies involving different SC types (BMSCs, ADSCs, UC- MSCs, hAECs) highlight the inhibition of granulosa cell apoptosis as an important mechanism (77, 78, 80, 83), which could be further amplified by heat shock pre-treatment (77) or collagen scaffold addition (78).

Men-MSCs is an extremely interesting category of SC therapy. They constitute a stem cell population that gathers both endometrial stromal fibroblasts and perivascular eMSCs, anticipating a similar identity to eMSCs obtained from endometrial biopsies (38, 95, 96). Preclinical studies investigating human Men-MSCs transplantation in rodent models of POI shed light into novel mechanisms of ovarian function restoration, such as renewal of OSC pool (93), amelioration of fibrosis via downregulation of TGF-β1/SMAD 2,4 pathway (97) and secretion of fibroblast growth factor 2 (FGF-2) (98), upregulation of extracellular matrix (ECM)-dependent focal adhesion kinase (FAK)/Akt signaling pathway (99), and most importantly, their potential to differentiate into granulosa cells (100, 101) which is unique among MSCs. In conjunction with decreasing granulosa cell apoptosis, the latter underpins the restoration of normal follicle development (99) and hormonal function, which is observed not only in preclinical studies (97, 99, 100, 102), but also in the only, up-to-now, conducted clinical study in POI (94). Despite the improved ovarian markers (AMH, inhibin α/β, FSH receptor) (100, 102) and increased number of neonate births in rodents (93, 97), the respective human study neither shows increase in AMH nor reports clinical or biochemical pregnancies. However, it should be noted that autologous Men-MSCs transplantation has successfully increased pregnancy rates and LBR in patients with POR, with a significant percentage of them achieving natural conception (103). Considering the above findings together with the non-invasive process of harvesting Men-MSCs, this approach definitely deserves further evaluation in clinical studies.

Regarding potential risks of SC therapy, there are indeed few studies assessing safety. The primary concern is tumorigenicity, which is higher when hPSC are used (75). However, all the conducted clinical studies in POI have employed MSCs and no such events have been reported. However, all these studies, including one having safety as primary outcome (37) are not controlled and their follow-up is short (maximum 1 year) (33–37). On the other hand, regarding transplantation of adult MSCs, their differentiation and proliferative capacity significantly decrease with aging, limiting their efficacy (62) and survival, which might be as short as 4 weeks (104). Moreover, allogeneic transplantation carries the risk of immune rejection (62) as well as ethical issues, like the transfer of foreign DNA into the foetus; regarding the latter, both one study examining the transplantation of human BMSCs in POI mouse model and the only study using allogeneic UC-MSCs in women with POI is reassuring, showing no human DNA transfer into mouse foetuses (104), as well as that the human foetus is genetically related to the mother and not the donor (35). Finally, autologous SC transplantation carries less risk of immunogenicity and has no ethical concerns; however, it requires two levels of invasiveness (except for Men-MSCs). Considering all the above and given the high cost of the procedure, the clinical application of SC-therapy requires further validation through properly designed controlled, long-term, clinical trials.

## 6. Micro-RNAs

Micro-RNAs are short, 18–24 nucleotides long, non-coding RNAs, which regulate cell proliferation, differentiation and apoptosis (14). Their pathogenetic role in POI has been increasingly recognized over the last years, being involved in steroidogenesis, granulosa cell proliferation/apoptosis, autophagy and follicular development by regulating specific pathways, such as the PI3K/Akt/mTOR, TGFβ, mitogen-activated protein kinase (MAPK) and Hippo pathways (105). Many of these molecules have shown to be either upregulated or downregulated in models of POI and have been proposed as biomarkers (106). In particular, miR-122-5p, miR23α, miR146α and miR27α have been shown to induce granulosa cell apoptosis via B-cell lymphoma 9 (BCL-9), X-linked inhibitor of apoptosis protein (XIAP), caspase cascade pathway and SMAD5 respectively (106–108); moreover, miR-127-5p attenuates repair capability of granulosa cells via HMGB2 gene (109).

This understanding has stimulated research in order to investigate potential disease-modifying role of micro-RNAs in chemotherapy-induced mice models of POI, with so far, promising results. For example, miR-17-5p, derived from human UC-MSCs-exosomes, promotes the proliferation of damaged granulosa and ovarian cells and decreases oxidative stress, via inhibition of sirtuin 7 (SIRT7) expression (110). In addition, miR-29α promotes the proliferation of granulosa cells and suppresses their apoptosis, reserves the existing mature follicles and restores ovarian function, via targeting wingless-related integration (Wnt)/β-catenin pathway (111). Up to now, there are no clinical data about the use of microRNAs in patients with POI.

From the applications in experimental setups in mice models of POI, it appears that the treatment with miRNAs leads to granulosa cells proliferation and continuous improvement of ovarian function, however we do not yet have clinical data from the application of such treatment in women with POI.

## 7. Mitochondrial-targeting therapies

Mitochondrial dysfunction is mainly associated with ovarian aging (112, 113). In addition, patients with idiopathic POI have been demonstrated to have significantly less mitochondrial DNA content (114), to bear more mitochondrial mutations (115), especially in the respiratory chain, and to have higher reactive oxygen species level and lower adenine triphosphate level (115), in comparison to fertile, healthy women. These findings suggest that mitochondrial dysfunction is implicated in pathogenesis of POI.

Mitochondrial replacement therapy has been applied in women with low-quality oocytes of various aetiologies and not in POI populations. Allogeneic mitochondrial replacement therapy, i.e., the transfer of mitochondria from a young, healthy donor to pre-implantation embryo during IVF (116), has been banned by FDA in 2001 due to ethical concerns regarding the risk of heteroplasmy (113) and subsequently was made again legal in UK in 2015 for cautiously selected cases. Since 2019, ESHRE strongly discourages mitochondrial donation, due to lack of solid scientific evidence proving safety and efficacy (117). Unfortunately, the attempt to perform autologous mitochondrial transplantation from mitochondria of OSCs to embryo during intracytoplasmic sperm injection (ICSI), in a large-scale, triple-blind randomized controlled trial (AUGMENT), showed no increase in fertilization or euploidy rate, therefore the study had to be terminated (118).

Studies with nutrients targeting mitochondrial function, such as Q10, resveratrol and melatonin, reveal that the latter might be effective in delaying ovarian aging, via increasing antioxidant capacity, maintaining telomerase activity and activating sirtuin 1 (14). Finally, photobiomodulation therapy (PBMT) with low-level laser light therapy (LLLT) is known to exert its rejuvenative effects via targeting the chromophore cytochrome C oxidase in mitochondrial membrane (119). In two rodent studies, PBMT is shown to increase the number of primary and pre-antral follicle count (120, 121), to decrease granulosa cell apoptosis and to increase angiogenesis; however, it was not shown to increase the number of Pfs (120).

## 8. What is the efficacy of biologic therapies for POI in comparison to the standard-practice pharmacological (hormonal) treatments?

Women with POI have 5%–10% chance of spontaneous conception (4), due to maintenance of intermittent ovarian function (2). Those seeking fertility should receive a cyclic rather than a continuous HRT regimen, in order to optimize their chances (1). In women with untreated POI, increased level of gonadotropins induces a maladaptive hormonal feedback, in which the tonic rises of LH may lead to premature luteinisation of growing antral follicles (122) and increased FSH may down-regulate the FSH receptors in granulosa cells. Both phenomena minimize the chances of spontaneous ovulation (11). Despite the expected benefit in fertility outcomes by HRT via decreasing gonadotropin level, a randomized, controlled, crossover study failed to demonstrate any benefit in folliculogenesis, ovulation and spontaneous pregnancy rate, in HRT-treated patients vs. the non-treated (123).

In consideration of assisted reproduction techniques, there is only one randomized, placebo-controlled study in 50 women showing benefit in ovulation (32% vs. 0%) and pregnancy rate (16% vs. 0%) in those treated with ethinyloestradiol (EE) before ovulation stimulation. According to this study, lowering of FSH <15 iu/L by HRT before starting ovarian stimulation is vital in order to obtain successful outcomes (124). However, in another, not-controlled study, pre-treatment with conjugated estrogens or EE in 100 women with POI results in pregnancy rates per cycle of 5.2%, which is identical to the rate of spontaneous pregnancy in this population (125). Interestingly, immune-suppressing medications such as dexamethasone (126) and azathioprine (127) have shown to substantially increase ovulation and pregnancy rates in IVF and to allow spontaneous pregnancy respectively, reflecting the considerable prevalence of autoimmune aetiology in otherwise “idiopathic” POI patients, which was confirmed in the latter case report with azathioprine.

Unfortunately, all clinical studies assessing the efficacy of novel, biological therapies for POI are uncontrolled, and, therefore, direct comparisons cannot be made. Intraovarian PRP administration leads to spontaneous conception in 7.4%–10% of women with POI within 2–12 months in two cohort studies (24). This is non-negligibly higher than the reported rate of spontaneous pregnancy in this population, which is 5%–10% during their reproductive lifespan post diagnosis of POI; however, the corresponding clinical data are rather limited. Additionally, intraovarian PRP leads to ovulation rate of 64.8% and embryo formation rate of 26.4% during IVF (29), which exceed the respective rates in IVF with EE pre-treatment; yet again, these results originate from only one cohort study. Finally, IVA has led to embryo formation rate of 26.57% (31) and pregnancy rate of 7%–8% (13, 31), which is also above the rate of spontaneous pregnancy in this population. In contrast to the seemingly better efficacy of PRP and IVA against traditional treatment, there are only 2 cases of pregnancy post SC transplantation in the literature (35) and there is no study assessing its efficacy in IVF procedures.

## 9. Conclusions and future perspectives

POI is a condition of heterogenous aetiology affecting up to 3.7% of the female population worldwide. Despite that HRT improves its symptomatology and long-term health consequences, it cannot treat infertility, which has been described as the most devastating aspect of the disease. The reconceptualization of ovarian reserve as a dynamic, rather than static cell population, together with the observation that 50% of POI patients maintain Pfs in their ovaries, has guided research in investigating novel, biological strategies of ovarian rejuvenation. These include PRP, exos therapy, IVA, SC therapy, microRNAs, and mitochondrial targeting therapies.

Among the above experimental methods of ovarian rejuvenation, intraovarian PRP administration seems to be better studied, less invasive, and more efficacious, especially considering spontaneous conception (7.4%–10%). Furthermore, PRP and IVA appear to be quite effective in achieving IVF conception, with rates of 4% and 7%–8% respectively. However, IVA has not been extensively studied. Considering that POI patients are not suitable candidates for IVF treatment using their own oocytes, the above data suggest that both techniques may help these women to have genetically-related offspring. In contrast to SC-based therapies which are expensive, PRP and IVA protocols are low-cost approaches. Furthermore, SC transplantation has been mainly investigated in animal models; clinical data are scarce despite the rather high number of registered studies in ClinicalTrials.Gov (=18 studies). Keeping in view with the literature data in women with POI, the efficacy of SC transplantation in achieving pregnancy is limited (0–14.3%, 2 cases in total). Autologous mitochondrial replacement therapy has not been examined in POI patients; yet, it showed no benefit in patients with low oocyte quality. Emerging biological approaches such as exos and micro-RNAs appear to be safe and have promising disease-modifying results in preclinical models, but require further validation in clinical studies.

In conclusion, biological therapies in POI show promise but are still in their initial experimental stage. Regarding the already clinically applied techniques, PRP, IVA, SC transplantation therapies, the short-term follow-up of the conducted studies does not allow us to draw conclusions either about the duration of ovarian rejuvenation by each method or about safety. In addition, the comparison between studies can be problematic because of the lack of standardization protocols regarding PRP preparation and administration. Furthermore, the scarcity of data in SC-transplantation makes it is difficult to develop standardized protocols regarding which type of SC should be selected, or which interval of SC transplantation is required. Therefore, we need properly-designed, controlled clinical trials, in order to assess the efficacy, safety and reproducibility of these procedures. Finally, given that infertility is the only indication to embark on biological therapy for POI, the assessment of successfulness should be based on clinical fertility indices, such as achievement of clinical pregnancy and number of take-home babies, while ovarian reserve indices, such as AMH and AFC, could be also informative. The isolated improvement in hormonal levels, although desirable, cannot prove the success of such therapies as it can be achieved by conventional POI treatment with HRT.

According to ClinicalTrials.gov, there are 8 ongoing clinical trials regarding PRP application, 3 regarding IVA and 5 regarding SC transplantation with their results being anticipated in the next 2 years.

Carefully designed experimental and clinical studies will illuminate our understanding about the safety and efficacy of these new infertility treatments in POI.
